# Comparison of the Effects of Adipose Extracellular Matrix/Stromal Vascular Fraction Gel Injection and CO_2_ Fractional Laser on Atrophic Acne Scar in Asians Through a 24‐Week Prospective, Randomized, Split‐Face Study

**DOI:** 10.1111/jocd.70131

**Published:** 2025-03-20

**Authors:** Tao Zhao, Mengjiao Li, Junxia Wang, Jianzheng Liu, Jingyi Wei, Xin Liu, Chao Gao, Bing Li

**Affiliations:** ^1^ Department of Dermatology Xijing Hospital, Air Force Medical University Xi'an China; ^2^ Xi'an Medical University Xi'an China; ^3^ Department of Cardiology Xijing Hospital, Air Force Medical University Xi'an China

**Keywords:** adipose extracellular matrix/stromal vascular fraction gel, atrophic acne scar, CO_2_ fractional laser

## Abstract

**Background:**

Adipose extracellular matrix/stromal vascular fraction gel (ECM/SVF‐gel) contains adipose‐derived stem cells, extracellular matrix, and other cell components, and possesses the ability to promote collagen production and serve as a filling agent.

**Aim:**

To assess the efficacy and safety of ECM/SVF‐gel injection for the treatment of acne scars, compared to CO_2_ fractional laser (CO_2_FL).

**Methods:**

We performed an open‐label, investigator‐initiated, assessor‐blinded, split‐face trial in Xijing Hospital, China, between July 11, 2020, and December 30, 2022. Patients exhibiting moderate to severe acne scars were randomly assigned to a single ECM/SVF‐gel injection on one half of the face or two sessions of CO_2_FL treatments on the other half. The primary outcome was the change in total Echelle d'Evaluation Clinique des Cicatrices d'acne (ECCA) score from baseline to 24‐week follow‐up. Secondary outcomes included the changes in the volume of scars assessed by the Antera 3D software.

**Results:**

A total of 11 participants were enrolled, and 10 completed the follow‐up. The mean age of patients was 27.5 ± 4.2, 7 female and four male, seven with Fitzpatrick skin Type III and four with Type IV. At 24weeks, the mean change in ECCA score was −60.25 on the side of ECM/SVF‐gel injection and − 43.25 on the side of CO_2_FL treatment (difference:−17.00 [95% CI: −24.56 to −9.44], *p* < 0.001). Antera 3D photography analysis showed that the mean change in scar volume was −33.17% on ECM/SVF‐gel injection and −19.69% on CO_2_FL (difference: −13.48% [95% CI:−22.16% to −4.79%], *p* = 0.004).

**Conclusions:**

ECM/SVF‐gel injection is an effective and safe approach in the treatment of acne scars.

**Trial Registration:**

ClinicalTrials.gov number: NCT06116162

## 1 Introduction

Acne vulgaris is a prevalent skin disease affecting adolescents and young adults. The facial region is mostly affected, with many individuals developing different degrees of atrophic acne scars. The presence of a permanent acne scar has been identified as a potential risk factor contributing to negative self‐perception, unemployment, and psychological distress [1].

Atrophic acne scar is caused by the loss of collagen and destruction of pilosebaceous units in the dermis. Various treatment options are available for acne scars, including fractional lasers, micro‐plasma, fractional radiofrequency, picosecond laser, chemical peeling, needling, and punch excision [2]. Fractional lasers represent the most frequently employed technique for the treatment of acne scars among the various modalities available. It involves the rapid heating and vaporization of skin tissue, which triggers a wound‐healing response, leading to the deposition of new collagen and elastin fibers by fibroblasts. However, patients with moderate to severe acne scars often require multiple sessions of laser therapy. It is common for Asian patients to experience adverse reactions, such as erythema, sensitive skin, and hyperpigmentation after multiple laser treatments [3], posing a significant challenge in acne scar treatment.

Adipose extracellular matrix/stromal vascular fraction gel (ECM/SVF‐gel) is derived from the skin adipose tissue. The formulation of the ECM/SVF‐gel excludes most of the lipids and tumescent fluid while being rich in adipose‐derived stem cells (ASCs), endothelial cells, and other cell components [4]. Previous studies have indicated that ASCs have the ability to secrete various types of collagens and stimulate collagen synthesis in fibroblasts. Unlike conventional fat grafting, ECM/SVF‐gel contains fewer lipid droplets and therefore does not cause significant swelling [4]. In contrast to an adipose‐derived stromal vascular fraction (Ad‐SVF), ECM/SVF‐gel is a solid substance and can be harvested efficiently within a timeframe of approximately 5 min from Coleman fat [5]. It has been reported that the local application of ECM/SVF‐gel can effectively enhance wound healing [5]. Furthermore, ECM/SVF‐gel injection has proven effective in rectifying the tear trough deformity and palpebromalar groove [6].

In the current study, we aimed to evaluate the efficacy and safety of ECM/SVF‐gel injection for treating acne scars and compare it with CO_2_ fractional lasers (CO_2_FL) through a prospective, randomized split‐face study.

## 2 Methods

### 2.1 Study Design

The current study was an open‐label, investigator‐initiated, assessor‐blinded, randomized split‐face clinical trial conducted in Xijing Hospital, China, between July 2020 and December 2022. This research received approval from the Institutional Review Board and was carried out in compliance with the Declaration of Xijing Hospital. Informed consent was secured from all participants involved.

### 2.2 Patient Population

Individuals aged 16–40, having a skin type of Fitzpatrick III‐IV, and exhibiting moderate to severe acne scars were considered eligible for inclusion. Individuals with moderate to severe acne scarring were classified based on Echelle d'Evaluation Clinique des Cicatrices d'acne (ECCA) scores [7] exceeding 50. All participants were prohibited from utilizing any systemic, topical, or laser therapies for acne and acne scars during the entire course of this study. The criteria for exclusion encompassed individuals with mental health disorders, those who were pregnant, participants who had utilized oral isotretinoin in the preceding 3 months, and individuals who had undergone laser treatments or other forms of chemical peeling within the last 8 weeks.

### 2.3 Randomization and Follow‐Up

Eligible patients were randomly assigned to a single ECM/SVF‐gel injection on one half of the face or two sessions of CO_2_FL treatments administered at intervals of 8 weeks on the other half. The treatment modality for each side was assigned through a randomization sequence generated by computer‐based random number generators. Given the open‐label design, the investigators and patients enrolled were not blinded to treatment allocation; however, dermatologists who adjudicated the endpoints, analysts who performed the quantitative analyses, and statisticians who developed statistical programs were blinded to the treatment allocation. Follow‐up visits were scheduled at 1, 8, and 24 weeks (±2 weeks) after randomization. Follow‐up appointments were ideally carried out; however, if patients were unable or unwilling to attend the outpatient clinic, the scheduled visit could be substituted with a telephone consultation, with the exception of the visits at 8 and 24 weeks.

### 2.4 ECM/SVF‐Gel Injection and CO_2_ Fractional Laser Treatment

Details on autologous fat graft harvesting were described in the Data S1. The formation of ECM/SVF‐gel has been reported previously reported [4]. In brief, the fat graft was subjected to centrifugation at 1200 rpm for a duration of 3 min, and the bottom layer of the tumescent fluid was removed. The Coleman fat, which was located in the upper layer, was subsequently collected and transferred between two 20 mL syringes that were linked via a Luer‐Lok connector with an internal diameter of 1.0 mm. This process involved six to eight transfers at a flow rate of 20 mL/s, resulting in the formation of a uniform emulsion of the Coleman fat. Then, it was sharked under negative pressure and processed through centrifugation at 2400 rpm for 3 min. Finally, the substance in the middle layer was ECM/SVF‐gel (Figure S1). The description of the ECM/SVF‐gel injection procedure and the immediate appearance following the treatment (Figure S2) were outlined in the Data S1.

Details of CO_2_FL treatment were also presented in the Data S1. In brief, the Acupulse device (LUMENIS, US) was used for CO_2_FL treatment. The laser was utilized in DeepFX mode, characterized by energy intensities ranging from 20 to 25 MJ, a coverage of 5%, an emission frequency of 300 Hz, and a spot size of 10 mm without overlap. Superficial mode was employed with energy intensities between 80 and 120 MJ, a coverage of 40% to 60%, an emission frequency of 300 Hz, and a spot size of 10 mm, also without overlap. The immediate appearance following the treatment was shown in Figure S2.

### 2.5 Outcomes Assessment

All participants were captured using a uniform digital camera (EOS 600D, Canon, Japan) under matching lighting conditions at each visit.

At both the baseline and the final follow‐up visit, VISIA digital photography (Visia CR; Canfield Imaging Systems, USA), 3D camera, and its associated software (Antera 3D CS, Miravex, Ireland) were utilized. The efficacy of scar improvement was evaluated using the ECCA score [7] during each appointment. The ECCA scores were rated by two skilled dermatologists who were blinded to the treatment allocation solely based on the photographs.

The primary outcome was the change in ECCA score from baseline to 24‐week follow‐up. Secondary outcomes include the assessment of alterations in the volume of atrophic scars, volume of skin pores, and roughness of the skin surface in the selected area, which were evaluated by Antera 3D software [8]. The patient‐reported outcome was recorded at the final follow‐up visit. The level of patient satisfaction is evaluated by a grading scale as follows: 0 indicates no improvement; 1 indicates 1%–25% improvement; 2 indicates 26%–50% improvement; 3 indicates 51%–75% improvement; 4 indicates 76%–100% improvement [9].

All Common Terminology Criteria for Adverse Events (CTCAE Version 5.0) defined events were recorded. Procedural‐related safety endpoints, including erythema, oedema, and dryness, all graded by a 0 to 3 severity scale (0 = none, 1 = mild, 2 = moderate, and 3 = severe), hyperpigmentation, and secondary scarring, were recorded [10].

### 2.6 Sample Size Determination and Statistical Analysis

The sample size estimation was based on detecting a between‐group difference of 10 points in the change of ECCA score (chosen based on the clinically accepted threshold to show the difference between treatment modalities) [9], a standard deviation of 10 for both the ECCA scores measured at baseline and follow‐up, and the correlation between measurements of 0.8 (square root of the *R*‐squared between the two measurements). Anticipating a 10% dropout rate, 18 samples (9 patients) would provide 80% power at a 2‐sided *α* of 0.05.

For the primary endpoint, the analysis of group comparisons was conducted utilizing mixed‐effect models by fitting the interaction between treatment groups (ECM/SVF‐gel injection or CO_2_FL treatments) and time point as fixed effects and patient identity as the random effect. These models account for repeated measures (baseline and follow‐up) and for the correlation of two sides of the face per patient. For the secondary endpoints, paired *t*‐tests were used to compare the change in the volume of atrophic scars, skin pores, and skin surface roughness between the two treatments. The Wilcoxon signed‐rank test was used to compare the group difference in patient satisfaction grade. The Friedman rank sum test was employed to assess the differences in safety endpoints across the groups. A *p* value of < 0.05 was deemed statistically significant. The analysis was done with the R statistical software version 4.2.1 (R Project for Statistical Computing).

## 3 Results

### 3.1 Patient Characteristics

Between July 11, 2020, and December 30, 2022, 11 participants were enrolled and randomized. Patients' characteristics at baseline and their clinical presentation are shown in Table 1. Overall, the mean age of patients was 27.55 ± 4.2, with seven female and four male participants, seven categorized as Fitzpatrick skin Type III and four as Type IV. The total ECCA score at baseline was 148.25 ± 15.00 on the half face with ECM/SVF‐gel injection and 128.25 ± 16.33 on the other half with CO_2_FL treatment.

**TABLE 1:** Baseline characteristics.

Characteristics	SVF‐gel filling side	CO_2_FL side
Age, mean ± SD, year	27.5 ± 4.2	
Sex, *n* (%)		
Male	4 (36.4)	
Female	7 (63.6)	
Fitzpatrick skin type, *n* (%)		
Type III	7 (63.6)	
Type IV	4 (36.4)	
ECCA score, mean ± SD		
Total	148.25 ± 15.00	128.25 ± 16.33
V‐type	31.50 ± 8.51	29.25 ± 8.25
U‐type	48.00 ± 10.33	44.00 ± 10.75
M‐type	68.75 ± 12.15	55.00 ± 15.81

### 3.2 Primary and Secondary Outcomes

**FIGURE 1:**
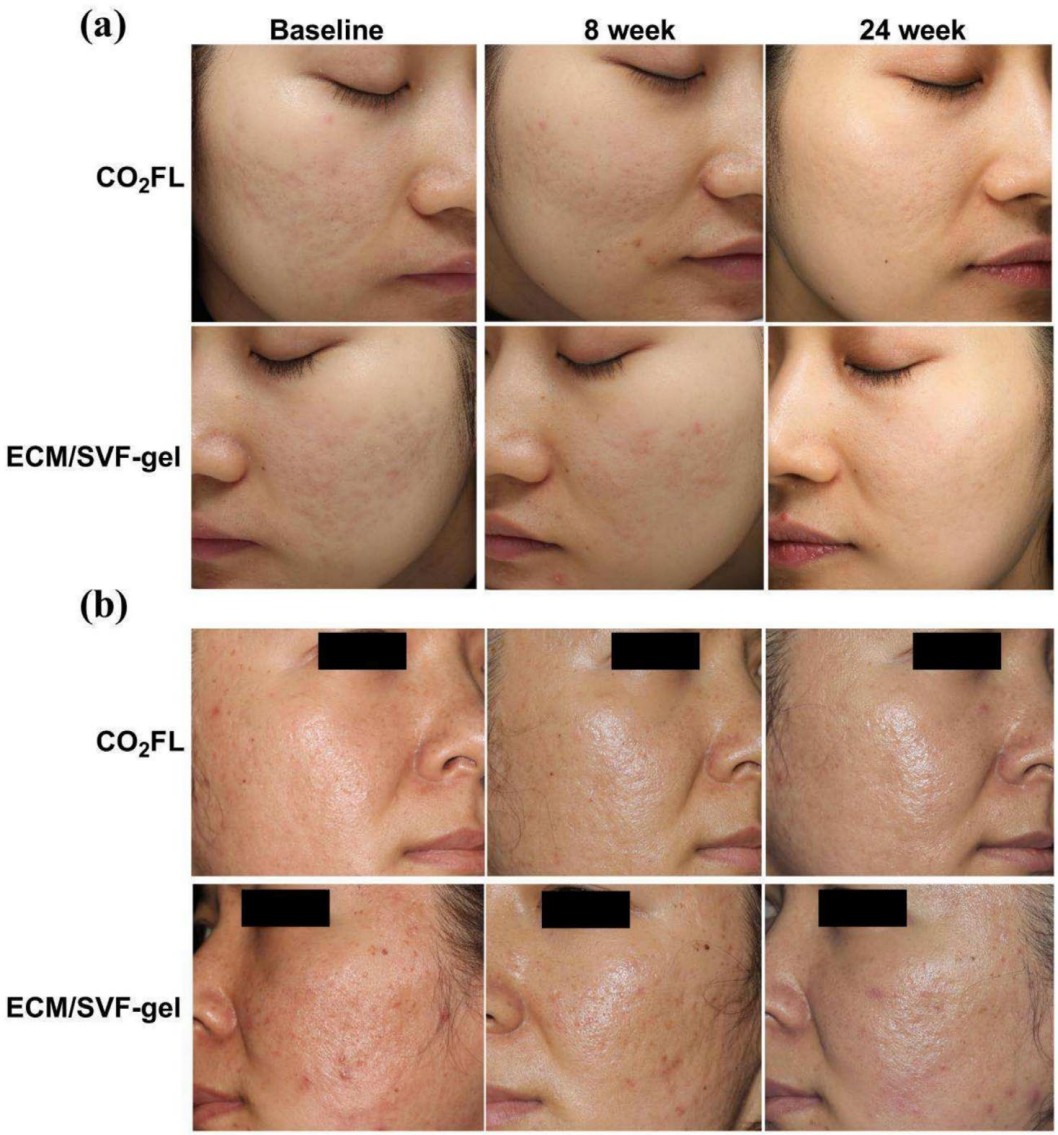
Clinical photographs demonstrated an enhancement in the appearance of acne scars. (a) VISIA digital photographs of a 27‐year‐old female with M‐shaped scars, V‐shaped scars, and pigmentation at baseline, 8 weeks after ECM/SVF‐gel injection on the left side of the face and CO_2_FL treatment on the right side of the face, and 24 weeks after second treatments of CO_2_FL on the right side of the face and no treatment on the left side of the face; (b) Camera photographs of a 26‐year‐old female with severe mixed‐type acne scars at baseline, and the treatment design was identical to the one mentioned above. CO_2_FL, CO_2_ fractional laser; ECM/SVF‐gel, Adipose extracellular matrix/stromal vascular fraction gel injection.

Of all 11 participants enrolled, 10 completed the study, and seven completed VISIA and Antera 3D photography. The primary endpoint, the change in mean ECCA score from baseline, showed a significantly greater reduction in the ECM/SVF‐gel injection group compared to the CO_2_FL treatment group (−60.25 [95% CI: −70.42 to −50.08] vs. −43.25 [95% CI: −50.55 to −35.95]; between‐group difference, −17.00 [95% CI: −24.56 to −9.44], *p* < 0.001) (Tables 1 and 2; and Figure 1).

**TABLE 2:** Primary endpoint.

	ECM/SVF‐gel	CO_2_FL	Difference
Total ECCA score			
Baseline	148.25 ± 15.00	128.25 ± 16.33	20.00 ± 13.18
8 weeks FU	105.75 ± 21.70	95.00 ± 12.86	10.75 ± 17.00
24 weeks FU	88.00 ± 19.29	85.00 ± 15.28	3.00 ± 7.98
Change from baseline to 8 weeks (95% CI)	−42.50 (−50.89, −34.11)	−33.25 (−40.55, −25.95)	−9.25 (−16.81, −1.69)
*p*	< 0.001	< 0.001	0.028
Change from baseline to 24 weeks (95% CI)	−60.25 (−70.42, −50.08)	−43.25 (−50.55, −35.95)	−17.00 (−24.56, −9.44)
*p*	< 0.001	< 0.001	< 0.001
*p* for trend			< 0.001
V‐shaped ECCA score			
Baseline	31.50 ± 8.51	29.25 ± 8.25	2.25 ± 8.70
8 weeks FU	27.00 ± 5.24	22.50 ± 6.12	4.50 ± 8.06
24 weeks FU	24.75 ± 7.12	17.25 ± 3.62	7.50 ± 7.91
Change from baseline to 8 weeks (95% CI)	−4.50 (−9.02, 0.02)	−6.75 (−11.45, −2.05)	2.25 (−2.35, 6.85)
P value	0.051	0.01	0.352
Change from baseline to 24 weeks (95% CI)	−6.75 (−12.65, −0.85)	−12.00 (−17.18, −6.82)	5.25 (0.65, 9.85)
*p*	0.029	< 0.001	0.039
*p* for trend			0.034
U‐shaped ECCA score			
Baseline	48.00 ± 10.33	44.00 ± 10.75	4.00 ± 5.16
8 weeks FU	30.00 ± 8.17	30.00 ± 9.43	0.00 ± 6.67
24 weeks FU	27.00 ± 9.49	24.00 ± 4.83	3.00 ± 4.83
Change from baseline to 8 weeks (95% CI)	−18.00 (−21.02, −14.98)	−14.00 (20.03, −7.97)	−4.00 (−8.82, 0.82)
*p*	< 0.001	< 0.001	0.123
Change from baseline to 24 weeks (95% CI)	−21.00 (−25.6, −16.94)	−20.00 (−25.84, −14.16)	−1.00 (−5.82, 3.82)
*p*	< 0.001	< 0.001	0.693
*p* for trend			0.700
M‐shaped ECCA score			
Baseline	68.75 ± 12.15	55.00 ± 15.81	13.75 ± 10.94
8 weeks FU	48.75 ± 16.08	42.50 ± 13.44	6.25 ± 14.73
24 weeks FU	36.25 ± 13.76	43.75 ± 10.62	‐ 7.50 ± 6.45
Change from baseline to 8 weeks (95% CI)	−20.00 (−27.54, −12.46)	−12.50 (−18.46, −6.54)	−7.50 (−14.68, −0.32)
*p*	< 0.001	0.001	0.057
Change from baseline to 24 weeks (95% CI)	−32.50 (−41.14, −23.86)	−11.25 (−17.85, −4.65)	−21.25 (−28.43, −14.07)
*p*	< 0.001	0.004	< 0.001
*p* for trend			< 0.001

*Note:* Data are presented as mean ± SD.

Abbreviations: CO_2_FL, CO_2_ fractional laser; ECM/SVF‐gel, Adipose extracellular matrix/stromal vascular fraction gel injection.

Mean ECCA score reductions were also analyzed based on subtypes (V‐, U‐, and M‐shaped) of acne scars. Compared with CO_2_FL treatment, the ECM/SVF‐gel injection had significantly greater reductions in M‐shaped scars (between‐group difference, −21.25 [95% CI: −28.43 to −14.07], *p* < 0.001); no statistical between‐group difference was observed for the U‐shaped scars (between‐group difference, −1.00 [95% CI: −5.82 to 3.82], *p* = 0.693); while CO_2_FL treatment had greater reductions in V‐shaped scars than the ECM/SVF‐gel injection (between‐group difference, 5.25 [95% CI: 0.65 to 9.85], *p* = 0.039) (Table 1).

**FIGURE 2:**
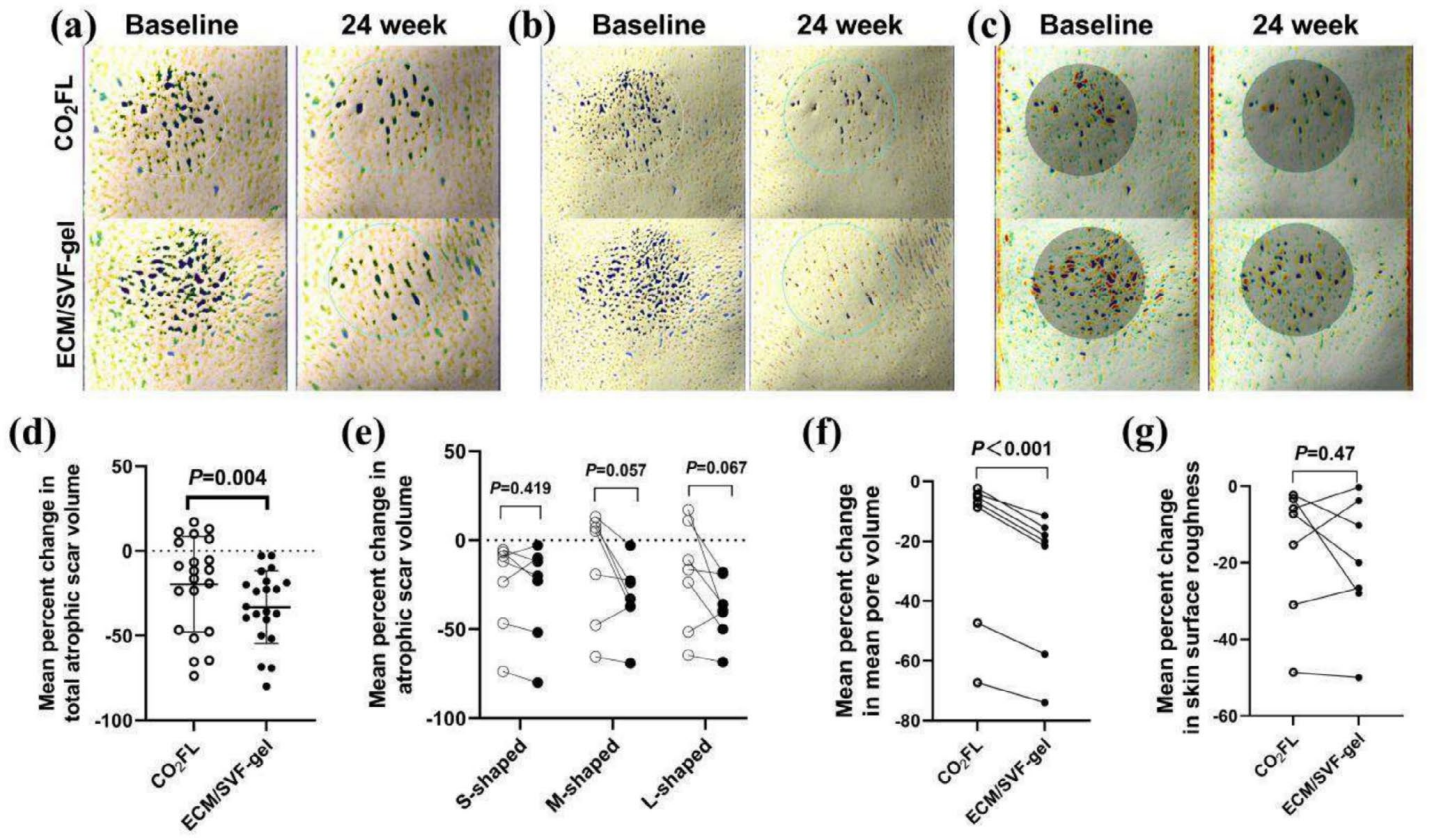
Three‐dimensional imaging assessments of atrophic scars, skin pore, and surface roughness of skin utilizing Antera 3D CS. (a–c) Presentative analyzed images of the sides of ECM/SVF‐gel injection and CO_2_FL treatment at baseline and 24 weeks. (a) The depths of atrophic scars and (b) skin pores (white < yellow < green < blue). (c) The vertical deviations of the skin surface (purple < blue < green < white < yellow < red). (d–f) Mean percent changes in (d) total atrophic scar volume, (e) subtype of atrophic scar volume, (f) mean pore volume, and (g) skin surface roughness. ECM/SVF‐gel, Adipose extracellular matrix/stromal vascular fraction gel injection; CO_2_FL, CO_2_ fractional laser; S‐shaped: Small‐shaped scars which diameter is 0.1–1 mm; M‐shaped: Medium‐shaped scars which diameter is 1.1–2 mm; L‐shaped: Lager‐shaped scars diameter is 2.1–3 mm.

Compared with the CO_2_FL group, the ECM/SVF‐gel group had a significantly lower atrophic scars volume (between‐group difference, −13.48% [95% CI: −22.16% to −4.79%]; *p* = 0.004) (Figure 2a,d) and skin pores volume (between‐group difference, −10.83% [95% CI: −13.42% to −8.23%]; *p* < 0.001) (Figure 2b,f). However, there was no difference between the two treatment groups in terms of mean change of sub‐type atrophic scars volume (Figure 2e) or skin surface roughness (Figure 2c,g).

**FIGURE 3:**
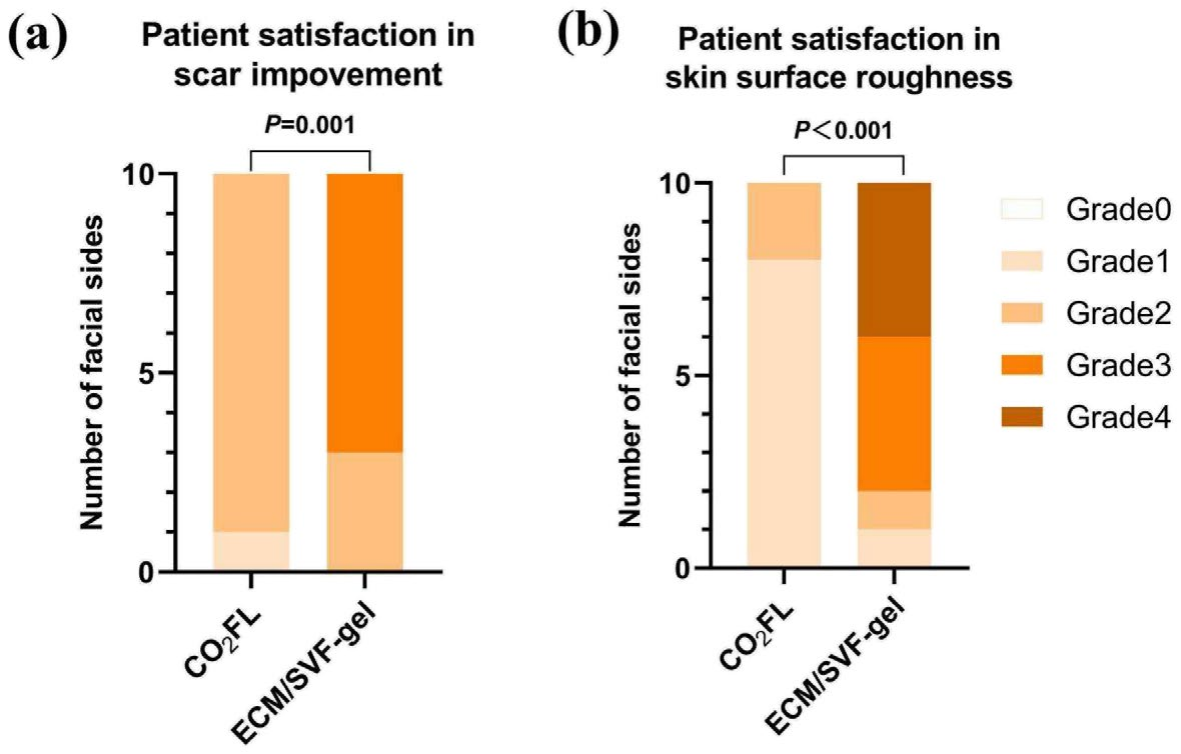
Patient satisfaction in the improvement of scar and skin surface roughness. The assessments were recorded at 24 weeks. ECM/SVF‐gel, Adipose extracellular matrix/stromal vascular fraction gel injection; CO_2_FL, CO_2_ fractional laser; Grade 0 = no improvement; Grade 1 = 1%–25% improvement; Grade 2 = 26%–50% improvement; Grade 3 = 51%–75% improvement; Grade 4 = 76%–100% improvement.

The patients' self‐reported satisfaction suggested that ECM/SVF‐gel injection group had significantly greater scar improvement (*p* = 0.001) and skin surface roughness (*p* < 0.001) (Figure 3). The patients' self‐reported preference suggested that six participants preferred ECM/SVF‐gel and four preferred CO_2_FL.

### 3.3 Safety Endpoints

**FIGURE 4:**
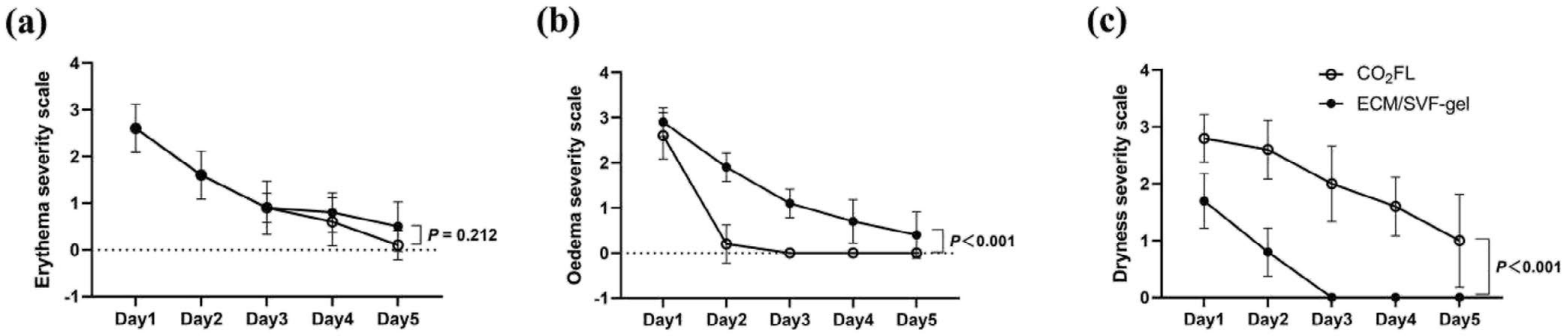
Evaluation of (a) erythema, (b) oedema, and (c) dryness on both sides for post‐treatment 5 days. ECM/SVF‐gel, Adipose extracellular matrix/stromal vascular fraction gel injection; CO_2_FL, CO_2_ fractional laser.

No CTCAE defined adverse event occurred during the study course. Adverse effects associated with the procedure, such as erythema, edema, and dryness, were noted in both treatment groups among all participants. Most of these symptoms were found to resolve within a span of 5 days. The severity of edema was greater in the ECM/SVF‐gel group compared to CO_2_FL (*p* < 0.001) (Figure 4b); conversely, the dryness is more pronounced in the CO_2_FL group (*p* < 0.001) (Figure 4c). No between‐group difference was observed for erythema (*p* = 0.212) (Figure 4a). There were no secondary scarring or infections. In the CO_2_FL treatment group, two cases had mild localized pigmentation.

## 4 Discussion

ECM/SVF‐gel represents an innovative injectable mixture produced through a straightforward mechanical method, characterized by a high concentration of ASCs, vascular endothelial cells, and the native ECM. In the current study, a comparative analysis was performed between ECM/SVF‐gel injection and CO_2_FL for the management of acne scars. The principal outcomes can be summarized as follows:

1. Both treatments exhibited improvement in the appearance of acne scars. Nevertheless, a single ECM/SVF‐gel injection showed a more significant enhancement compared to two sessions of CO_2_FL treatment, as evidenced by a greater reduction in ECCA score.
2. ECM/SVF‐gel injection exhibited superior effectiveness in treating M‐shaped scars, whereas CO_2_FL is better for V‐shaped scars.
3. In addition, compared with CO_2_FL, ECM/SVF‐gel injection can also improve skin pores.

CO_2_FL of 10,600 nm has been widely applied and remains the first‐line treatment for acne scar treatment [1]. Most scholars have used the CO_2_FL in deep mode combined with the superficial mode to treat acne scars and found an improvement rate of 25%–50% after two sessions of treatment without serious adverse reactions [11]. International consensus recommendation mentioned that 2–4 sessions of CO_2_FL treatment are recommended to achieve a satisfactory clinical response [1]. In this study, we also combined the two modes of Acupulse CO_2_FL to treat acne scars for two sessions. The results showed that ECCA scores were decreased by 33.5%.

Injection therapy presents a viable option for addressing acne scars through enhancing dermal tissue loss and adhesion. Various studies have highlighted the effectiveness of different injection methods such as polymethylmethacrylate injection [12, 13], hyaluronic acid injection [14, 15], intradermal injection of hyaluronic acid and abobotulinumtoxin A mixture [16], autologous plasma gel injection [17], autologous fibroblast injection [18, 19], autologous bone marrow stem cells [20] and platelet‐rich plasma injection [21]. Nevertheless, there is a gap in research regarding the comparison between injection therapy and fractional laser treatment for acne scars.

Adipose‐derived stromal vascular fraction (Ad‐SVF) isolated from autologous adipose tissue contains ASCs, vascular endothelial progenitors, macrophages, fibroblasts, smooth muscle cells, and various blood cells. In recent years, Ad‐SVF has been used to improve alopecia [22], wound healing, and tissue regeneration [23]. Two techniques are available for isolating Ad‐SVF: mechanical methods and enzymatic methods. Enzymatic methods are commonly used for high effectiveness in obtaining a larger quantity of ASCs compared to mechanical methods [24]. In a previous study, local application of Ad‐SVF‐derived exosomes or conditioned media combined with CO_2_FL was used to treat acne scars [10, 25]. Shan et al. [26] found that subcutaneous injection of ASCs ameliorated acne scars and reduced inflammation in animal models of acne scars. Behrangi et al. [27] found that subcutaneous injection of nanofat and Ad‐SVF improved acne scar more significantly than nanofat injection. Despite the fact that Ad‐SVF is rich in ASCs and has the ability to improve scars, the process of isolating it is complex. Occasionally, it becomes necessary to employ in vitro cultivation and expansion techniques in order to obtain a sufficient quantity of Ad‐SVF.

ECM/SVF‐gel is, which selectively destroys and eliminates most mature adipocytes using a simple mechanical method, and is rich in ASCs and native ECM [4]. In contrast to Ad‐SVF, it possesses the ability to not only induce collagen production but also has the capacity to serve as a filling agent [5]. In addition, unlike conventional fat grafting, ECM/SVF‐gel grafting contains little lipid droplet and does not result in significant swelling [6]. Previous studies suggested that the application of ECM/SVF‐gel can significantly accelerate wound healing [5] and improve wrinkles [6]. In this study, we used ECM/SVF‐gel injection to treat acne scars and had satisfactory effect. We found that compared to CO_2_FL, ECM/SVF‐gel injection is better for amelioration of M‐shaped scar. Importantly, our results demonstrate that a single ECM/SVF‐gel injection resulted in a consistent improvement of acne scar over a period of 24 weeks.

Previous studies showed that CO_2_FL are more effective in treating M‐shaped and U‐shaped scars than V‐shaped scars [1, 3]. Our results suggested that ECM/SVF‐gel injection is better for the improvement of M‐shaped scars than CO_2_FL. However, for U‐shaped scars, these two treatments showed no difference, and CO_2_FL is more effective for treating V‐shaped scars than ECM/SVF‐gel injection.

CO_2_FL has been recognized as a safe and efficient option for improving enlarged skin pores [28]. Our findings have demonstrated that ECM/SVF‐gel injection surpasses CO_2_FL in terms of its effectiveness in improving skin pores. In addition, both treatments have the potential to enhance the roughness of the skin surface.

In individuals of Asian descent with Fitzpatrick skin Types III and IV, the occurrence of sensitive skin and hyperpigmentation is a prevalent adverse reaction following CO_2_FL treatment [29]. However, in contrast to CO_2_FL, ECM/SVF‐gel injection causes minimal damage to the epidermis, resulting in a lower risk of sensitive skin and hyperpigmentation. Consistently, our results demonstrate that the CO_2_FL group exhibits a higher degree of skin dryness compared to the ECM/SVF‐gel injection group. No instances of hyperpigmentation were observed in the ECM/SVF‐gel injection group, whereas two cases in the CO_2_FL group had mild localized pigmentation. Additionally, the severity of edema was greater in the ECM/SVF‐gel group compared to CO_2_FL; nevertheless, it was resolved within 5 days. Consequently, ECM/SVF‐gel injection may be more appropriate for patients with Fitzpatrick skin Types IV and V in the treatment of acne scars.

In this study, there are still some limitations. We evaluated the impact of ECM/SVF‐gel injection in comparison to Acupulse CO_2_FL therapy for the treatment of acne scars. In a previous study, the effectiveness of Ultrapulse CO_2_FL is better for acne scar treatment than Acupulse CO_2_FL [4]. Furthermore, due to the COVID‐19 pandemic in China during the course of the study, the enrollment process has been significantly delayed, resulting in a limited sample size. Thus, there is still a need further study to compare the effectiveness of ECM/SVF‐gel injection to CO_2_FL in the treatment of acne scars.

## 5 Conclusion

In conclusion, acne scars are various regarding the shape and depth. It is still a challenge to reach satisfactory results. In this study, we compared ECM/SVF‐gel injection to CO_2_FL in the treatment of acne scars. We found that these two methods can ameliorate acne scars. ECM/SVF‐gel injection is more suitable for M‐shaped scars, and CO_2_FL is more suitable for V‐shaped scars. Thus, sequential therapy of CO_2_FL in combination with ECM/SVF‐gel injection may be a better choice for the treatment of severe mixed‐type acne scars.

## Author Contributions

Bing Li and Tao Zhao were responsible for the study design. The ECM/SVF‐gel injection was carried out by Tao Zhao and Bing Li, while Xin Liu administered the CO_2_FL treatment. Mengjiao Li, Junxia Wang, and Jingyi Wei were in charge of evaluating the ECCA scores, as well as conducting the photograph, VISIA, Antera 3D, and follow‐up procedures. Sample Size Calculation and Statistical Analysis were performed by Chao Gao and Jianzheng Liu. Bing Li wrote the manuscript. All authors contributed to the data interpretation, critically reviewed the manuscript, and approved the submitted version.

## Ethics Statement

This study was conducted in accordance with the Declaration of Xijing Hospital and was approved by the Institutional Review Board.

## Consent

All participants provided their written informed consent.

## Conflicts of Interest

The authors declare no conflicts of interest.

## Supporting information


Data S1.

## Data Availability

The data that support the findings of this study are available from the corresponding author (Bing Li) upon reasonable request.
