# Supplementary material for: Comparison of the Effects of Adipose Extracellular Matrix/Stromal Vascular Fraction Gel Injection and CO2 Fractional Laser on Atrophic Acne Scar in Asians Through a 24‐Week Prospective, Randomized, Split‐Face Study
Source: J Cosmet Dermatol. 2025 Mar 20;24(3):e70131. doi: 10.1111/jocd.70131 (PMC11925327; doi:10.1111/jocd.70131)
Supplement: Supplementary file 1 — Data S1. [file JOCD-24-e70131-s001.docx]

**Supplementary materials**

**Methods**

**Autologous fat graft harvesting**

The autologous fat graft was harvested from the lower abdomen or inner thigh of all participants. After local anesthesia, incisions with the size of 5 to 10 mm were performed on the margin of pubic hair for lower abdomen fat graft harvesting or on the groin for inner thigh fat graft harvesting. Then, the tumescent solution is equably infiltrated to the subcutaneous fat layer of donor sites through the incision using the blunt infiltration cannula. Knead the donor sites for 5-10 minutes to make harvesting of fat grafts easier to operate with less trauma and less painful. Next, the infiltration cannula used for liposuction was inserted into the subcutaneous fat layer of donor sites through the incision, and 20-mL Luer-Lok syringe is connected with a harvesting cannula with a 2.5-mm inner diameter. Gentle pulling back on the plunger creates a space vacuum negative pressure in the syringe. With gentle back and forth movement of the assembled syringe, the fat is gradually collected. After harvest, all incision sites are closed with interrupted sutures once excess tumescent fluid is milked out. Stretch bandage are routinely wrapped over the donor sites.

**ECM/SVF-gel injection**

0.1% lidocaine with 1:1000000 epinephrine is injected into the lesion, and adequate compression was applied to reduce swelling. The prepared ECM/SVF-gel was transferred to a 1-mL Luer-Lok syringe for injection. We used a 22G sharp-tip injector to inject the ECM/SVF-gel into the dermis and subcutaneous tissue of acne scars. A cold compress was used to minimize discomfort and swelling for the first 6 hours.

**CO_2_ fractional laser treatment**

For local anesthesia, a topical eutectic mixture of 2.5% lidocaine hydrochloric acid and 2.5% prilocaine (AstraZeneca AB, Sodertalje, Sweden) was applied to the half face under occlusion 30 minutes before treatments. Acupulse device (LUMENIS, US) was used for CO_2_ fractional laser treatment. The laser was operated in DeepFX mode under the parameters of 20-25 MJ energy intensity, 5% coverage, 300 Hz emission frequency and on the 10 mm spot size without overlap, Superficial mode under the parameters of 80-120 MJ energy intensity, 40-60% coverage, 300 Hz emission frequency, 10 mm spot size without overlap. As post-operative regimen, all participants applied facial cooling mask just after finishing the procedure for 15 minutes.

**
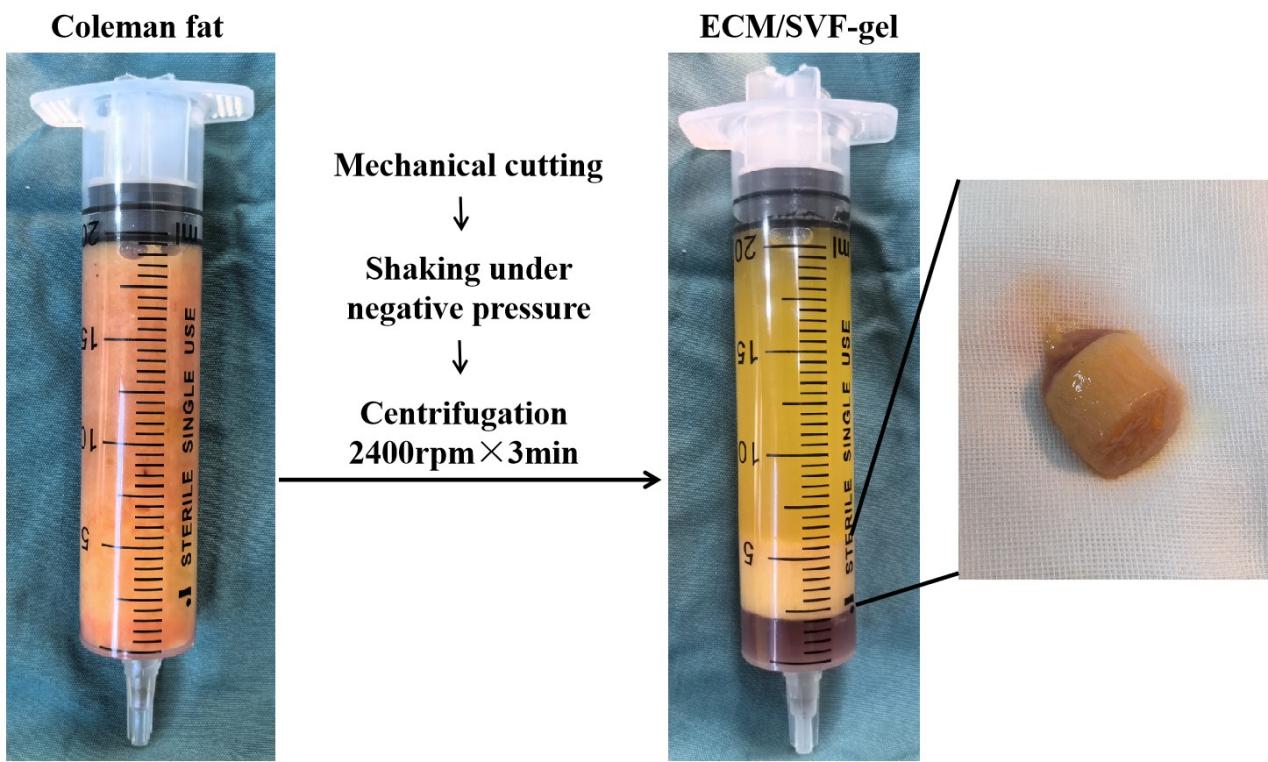
Supplementary Figure 1** The fabrication of ECM/SVF-gel.

**
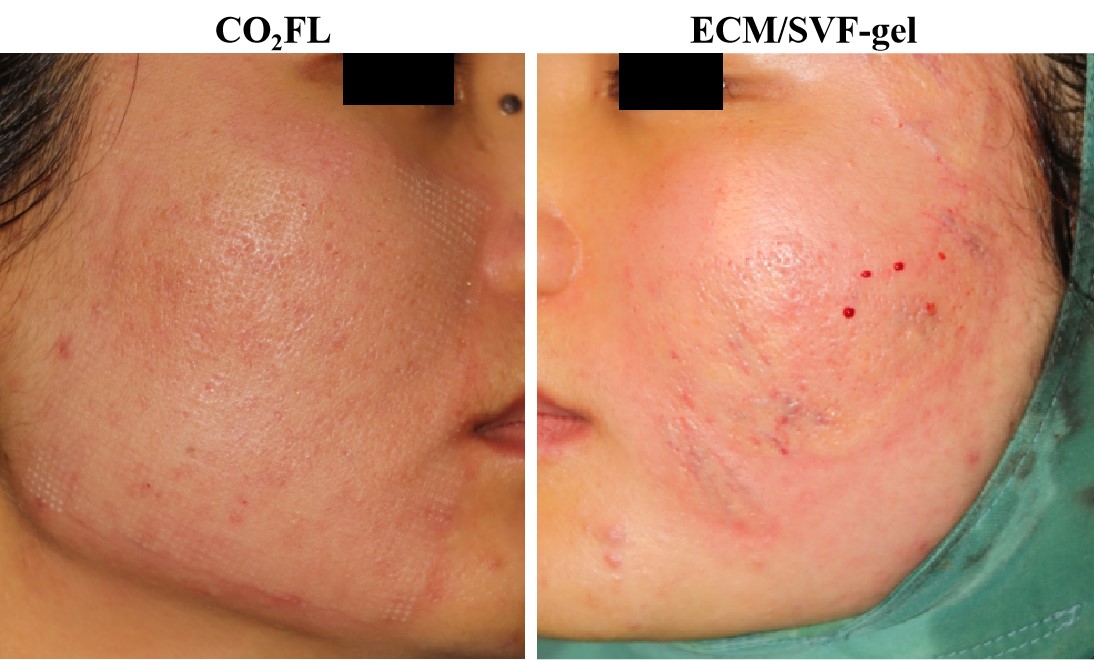
Supplementary Figure 2** Clinical photographs showed the instantaneous response following ECM/SVF-gel injection and CO_2_FL treatment. ECM/SVF-gel, Adipose extracellular matrix/stromal vascular fraction gel injection; CO_2_FL, CO_2_ ablative fractional laser.
